# Small molecule inhibition of RNA binding proteins in haematologic cancer

**DOI:** 10.1080/15476286.2024.2303558

**Published:** 2024-02-08

**Authors:** Amit K. Jaiswal, Michelle L. Thaxton, Georgia M. Scherer, Jacob P. Sorrentino, Neil K. Garg, Dinesh S. Rao

**Affiliations:** a Department of Pathology and Laboratory Medicine, University of California, Los Angeles, CA, USA; b Department of Chemistry and Biochemistry, University of California, Los Angeles, CA, USA; c Jonsson Comprehensive Cancer Center, University of California Los Angeles, CA, USA; d Broad Stem Cell Research Center, University of California, Los Angeles, CA, USA

**Keywords:** RNA binding proteins, post-transcriptional gene regulation, drug discovery, Acute leukaemia, RNA splicing

## Abstract

In recent years, advances in biomedicine have revealed an important role for post-transcriptional mechanisms of gene expression regulation in pathologic conditions. In cancer in general and leukaemia specifically, RNA binding proteins have emerged as important regulator of RNA homoeostasis that are often dysregulated in the disease state. Having established the importance of these pathogenetic mechanisms, there have been a number of efforts to target RNA binding proteins using oligonucleotide-based strategies, as well as with small organic molecules. The field is at an exciting inflection point with the convergence of biomedical knowledge, small molecule screening strategies and improved chemical methods for synthesis and construction of sophisticated small molecules. Here, we review the mechanisms of post-transcriptional gene regulation, specifically in leukaemia, current small-molecule based efforts to target RNA binding proteins, and future prospects.

## 1. Background

Dysregulation of gene expression is central to many disease states and occurs at several levels of both transcriptional and post-transcriptional control. While transcriptional control and dysregulation have been studied extensively, the role of post-transcriptional mechanisms have only recently come into focus as a significant pathogenetic mechanism. These mechanisms prominently involve the activity of RNA-binding proteins (RBPs), which are a functionally and structurally heterogeneous class of proteins that are only unified by their ability to bind to RNAs. RBPs control a range of processes that are important for the synthesis and homoeostasis of mRNA molecules. Moreover, RBPs have been reported to be dysregulated in multiple disease states, with pathologies typically manifesting due to altered expression levels. This type of dysregulation lends itself to pharmaceutical targeting, particularly in cases where RBPs are overexpressed. A significant amount of effort has been expended in targeting RBPs via oligonucleotide or other nucleic acid-based techniques, with some notable successes particularly in neurological diseases [1,2]. Here we focus on the current state of therapeutically targeting of RBPs with small molecules.

## 2. RNA-binding proteins regulate a range of RNA homoeostatic mechanisms within the cell

Within the cell, there are three main RNA polymerases that transcribe RNA from DNA [3]. RNA polymerase I is primarily responsible for the transcription of ribosomal RNA, RNA polymerase II is the enzyme that transcribes the bulk of messenger RNA (mRNA) and RNA polymerase III is responsible primarily for transcribing short RNAs such as transfer RNAs and small nucleolar RNAs. Of these polymerases, RNA polymerase II is subject to regulation by DNA-binding proteins, such as transcription factors and epigenetic regulators. This interaction with DNA-binding proteins by RNA polymerase II is the first step in the regulation of gene expression. However, once the pre-mRNA is produced, it undergoes a series of processing steps, including splicing, capping and polyadenylation mediated by RBPs [4–6]. There are a number of RBPs that may participate in ‘folding’ or generation of RNA secondary structures (RNA helicases) [7]. mRNA molecules are subject to modifications, constituting so-called epitranscriptomic marks [8,9]. This latter process appears to be dynamic, with some proteins depositing the modifications (i.e. ‘writers’) and others that remove the modifications (‘erasers’), with N^6^-methyladenosine (m^6^A) being the most common epitranscriptomic mark, although more than a hundred have been catalogued [10]. The last step in gene expression is for the mRNAs to be translated in the ribosome into proteins, which themselves are subject to further modification by a variety of covalent modifications.

Mature, modified mRNA molecules can be bound by RBPs that regulate their stability and access to translational machinery within the cell. One important class of such RBPs are the Argonaute proteins, which together with accessory proteins constitute the RNA-induced silencing complex (RISC). The activity of RISC, in turn, is dependent on microRNAs, which are small RNA molecules that bind to mRNAs via sequence homology. These are generated from genes encoding miRNAs by RNA polymerase III-based transcription followed by a series of endoribonucleolytic processing steps. miRNA/RISC-based repression of mRNA stability and translation has been posited as one of the major mechanisms regulating post-transcriptional gene expression.

**Figure 1.**
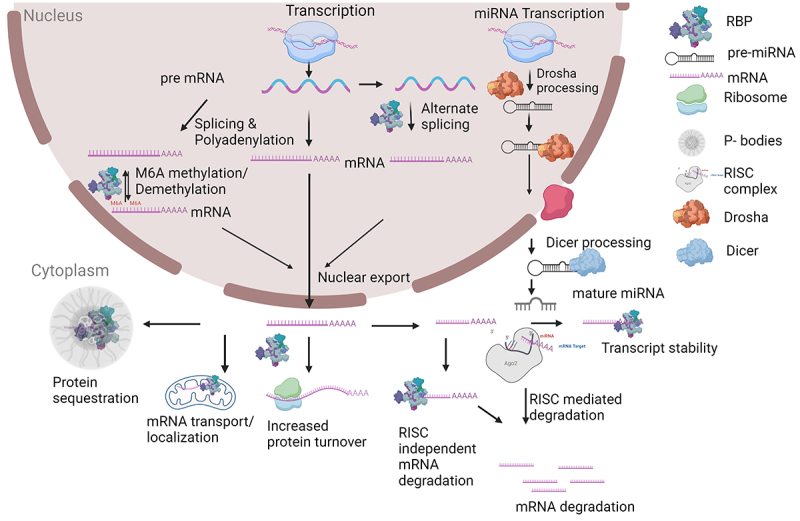
Schematic of RNA homoeostasis. Gene expression initates with transcription of DNA into a pre-mRNA. Numerous steps of RNA processing occur, many in parallel with transcription. A separate pathway of microRNA (miRNA) processing occurs for primary transcripts that encode miRNAs. Please see text for further details. Schematic created at Biorender.com.

From the preceding discussion, it should be apparent that RBPs can be characterized based on their cell biological function: splicing factors, capping enzymes, polyadenylation factors, RNA helicases, epitranscriptomic writers, erasers and readers. The function of RBPs can also be characterized as occurring via the regulation of RNA stability (RISC-dependent or -independent), or via the localization and transport of mRNA molecules (See Figure 1 for a summary of RNA homoeostasis). These different ways to conceptualize RBP function are overlapping and non-exclusive and are discussed in the chapters below. We also attempt to discuss novel potential therapeutics in light of these different conceptualizations of RBP function.

## 3. RBP dysregulation in cancer and leukaemia

RBPs show a range of dysregulation in cancer, including upregulation and downregulation as well as mutation [11]. These include proteins involved in a range of different RNA-related functions, and many functional studies have now documented a causal or supportive role in driving malignant transformation, including many of the hallmarks of cancer [12]. Splicing factors show altered expression and/or mutation in a range of haematologic malignancies [13], including acute myeloid leukaemia, myelodysplastic syndrome and chronic lymphocytic leukaemia, as well as in solid tumours. Experimentally, the expression of mutant splicing factors has been shown to recapitulate some features of disease, such as with the expression of point-mutated SF3B1 and SRSF2 in myelodysplastic syndrome [14,15]. In addition to splicing factors, RBPs that are involved in epitranscriptomic modifications have been shown to play a role in cancer, such as the m^6^A writer METTL13 and the m^6^A eraser FTO, which play functional roles in acute leukaemia [16,17]. Readers of these same modifications, such as the YTHDF2 and IGF2BP2 proteins seem to stabilize specific m^6^A-modified mRNA transcripts and promote their translation [18,19]. Further, several factors important in microRNA biogenesis, such as the LIN28 proteins, appear to modulate cancer causation [20]. Lastly, a number of proteins that are important in RNA stability are implicated in cancer causation. These include HuR (ELAVL1), which has been extensively studied and has an effect on both RISC-dependent and RISC-independent methods of mRNA stability [21–24]. IGF2BP3, which may also play a role in interpreting m^6^A signals, acts via an effect on mRNA stability, likely via the RISC [25–27]. However, it should be noted that some conflicting data exist as to the pro- or anti-oncogenic function of IGF2BP3 in specific subtypes of leukaemia [28]. Lastly, components of the translation initiation complex, e.g. eIF4E, have been shown to be overexpressed and functional in cancer progression and metastasis [29]. Together, these functional studies indicate the importance of RBPs and post-transcriptional pathways in driving cancer and indicate that they may represent good targets for cancer therapies.

## 4. Considerations for designing therapeutics against RBPs

Interestingly, the best functional data for RBPs’ specific role in cancer has been demonstrated for those RBPs which are overexpressed in cancer. In these functional studies, knockdown and/or knockout of the RBPs in a cancer driven by known oncogenes resulted in reduced intensity and/or elimination of various cancer characteristics. Given that chemical antagonism/inhibition may be more readily achievable than chemical agonism, we suggest that targeting RBPs with overexpression in cancer holds high promise [30–32]. The multiplicity of RNA targets bound by RBPs have led to the suggestion of ‘housekeeping’ functions for many of these proteins [33]. While there are clearly RBPs that have high basal expression in many tissues and may truly be ‘housekeeping genes’ with little differential expression in cancer, many RBPs show temporally and spatially restricted expression patterns, including some that demonstrate an oncofetal pattern. A careful analysis of existing data on RBP expression should be carried out to assess for a ‘therapeutic window’. Further, it has been suggested that RBPs that bind RNA in a sequence-independent manner appear to be associated with constitutive or housekeeping function, while those with sequence specific binding appear to have a more specific function [34]. Such sequence specific RBPs with temporal and spatial restricted patterns of expression may therefore be ideal candidates for cancer-specific inhibition. Hence, structure-function and structural analyses of the protein may also assist in understanding the requirements for RNA binding. In addition, high throughput analyses of RNA binding sites, such as those obtained from CLIP-seq, and identification of direct regulatory mRNA targets, by combining binding site data with differential expression analyses, may aid in designing specific assays to measure on-target activity of a therapeutic devised against a specific RBP. Various modalities have been used to target RBPs, including oligonucleotides, peptides, and small molecules. Each of these has distinct advantages and disadvantages; in the current article, we will review small molecule approaches. Small molecule therapeutics are a desirable modality over other strategies for targeting RBPs due to their generally favourable pharmacokinetic properties, the potential to be administered orally and to pass through cell membranes to reach intracellular targets. There are other reviews that more globally consider these alternative approaches [35,36].

## 5. Structural features of RBPs and considerations for targeting

The binding of RBPs may be dependent on primary sequence, secondary structure, as well as epigenetic modification (e.g. m^6^A) of the mRNA molecules. Many RBPs are characterized by containing one or more RNA-binding domains (RBDs) that bind to specific RNA sequences and structural motifs. The most well-defined and prevalent RBDs are the RNA recognition motif (RRM), hnRNP K homology (KH), DEAD/DEAH helicase and zinc-finger domains [37]. However, many RBPs lack these domains, and the domains themselves do not necessarily confer a specific mRNA sequence that is bound. Furthermore, many RBPs exist in large multiunit complexes, as has been well demonstrated for the splicing factors that work together in the spliceosome [4]. Classically, RBPs were considered undruggable, as most lack enzymatic activity that provides a handy read-out for measuring on-target activity [30]. Rational design of small molecules was also considered to be difficult, given the lack of highly specific structural motifs for intermolecular interactions or binding pockets within RBPs. Indeed, combinatorial recognition of target RNA sequences may be the rule for many RBPs, as has been demonstrated for IGF2BP3, rendering it difficult to design a small molecule that can target the binding sites [38]. Additionally, in vivo data to demonstrate the anti-cancer effect of RBP loss-of-function was previously lacking for many proteins. These features hindered the development of small molecule-based approaches to targeting RBPs. Recently, however, the advent and refinement of high-throughput screening approaches have allowed for the identification of small molecule inhibitors of RBPs which were once considered undruggable. We first briefly review some of these approaches before discussing specific inhibitors of RBPs.

## 6. High throughput screening approaches to developing RBP-targeted small molecule therapeutics

Multiple strategies have been used for high throughput screening to discover inhibitors of RBPs. In this section we discuss some commonly used screening techniques in drug discovery.

### 6.1. F ӧrster resonance energy transfer (FRET) based assay

FRET based methods are powerful tools and a highly efficient platform for drug discovery [39]. The assay is dependent on the overlap of fluorescence emission spectrum of a donor dye, linked to RNA, with the excitation spectrum of an acceptor dye, linked to an RBP, and physical proximity of the RBP and bound RNA [40]. Binding of a small molecule inhibitor with the protein restricts the binding of the RNA ligand; as a result, there is a gain/loss of endpoint fluorescence signal depending on the assay type. For example, a small molecule inhibitor SB1301 (**10.4**, Figure 4) of the LIN28 protein, which regulates miRNA biogenesis, was identified using the FRET-based method [41].

### 6.2. Fluorescence polarization (FP) assay

This method has been widely used for the high throughput screening of compounds for drug discovery. The method is homogenous, sensitive, robust and relatively insensitive to certain types of technical interference [42]. The principle of FP is that changes in the apparent molecular weight of the probe (in this case, a labelled nucleic acid) alter the polarization of the sample’s emitted light. In the unbound state, rotational diffusion of the probe leads to depolarization of light; when bound (to a RBP in this case), the apparent molecular weight increases and leads to decreased rotational diffusion and a relative increase in emission of polarized light. An example of small molecule inhibitor identified by FP was C902 (**10.6;** Figure 4), again targeting LIN28 [43].

### 6.3. Amplified Luminescent Proximity Homogeneous Assay (ALPHA) Screen method

ALPHA screen is a bead-based method that is dependent on the interaction of biological molecules. The endpoint fluorescence is dependent on the proximity of the two biological molecules. The singlet oxygen from the donor molecule is excited upon photosensitization and diffuses across to react with thioxene derivative of the acceptor beads, emitting a fluorescent light at 520–620 nm wavelength range. Although amenable to high throughput screening, it has been more extensively used in confirmation of small molecule interference with RNA-RBP binding, as in the case of the poly-A binding protein MSUT1 [44].

### 6.4. Firefly dual luciferase assay

The dual luciferase reporter assay is widely used in high throughput screening and is considered a ‘workhorse’ for drug discovery. The assay relies on a luciferase reporter fused to a functional RNA element that is under the control of the RBP under study. The reporter construct is introduced into cells, and luciferase activity is measured in the presence and absence of RBP and/or control reporter constructs. The screening strategy is directed at finding small molecules that return the luciferase reporter activity to control levels. This assay has been used to readout splicing and 3’UTR reporters. Specifically, the inhibitors NVS-SM1 and NVS-SM2 were developed against SMN2, an RBP involved in alternative splicing, using the firefly dual luciferase assay [45].

### 6.5. Catalytic enzyme-linked click-chemistry assay (cat-ELCCA)

The cat-ELCCA is based on click chemistry and has demonstrated applicability in studying biological processes and drug discovery. Based on development of click chemistry [46], this type of assay represents a new class of biochemical assay that involves bioorthogonally tagged analytes and enzymes, in a manner akin to ELISA. This type of assay has been used for HTS to identify small molecule inhibitors against LIN28 [47]. In this assay, a known RNA sequence labelled with 5’-trans-cyclooctene (TCO) interacts with immobilized LIN28 protein. The TCO can then undergo a ‘click’ reaction with methyltetrazine bound to horseradish peroxidase (HRP), bringing LIN28 and HRP into proximity, producing a chemiluminescent signal. In the presence of an RBP inhibitor, the TCO-bound RNA will not bind to LIN28 and chemiluminescence will not be observed. Small molecule inhibitors targeting LIN28, CCG-233094 and CCG-234459 (**10.7** and **10.8**; Figure 4) were identified using this method [47].

Each of these techniques represents a unique method to quantify the strength of the RBP-RNA interaction, with both biochemical and cellular-based methods described. Coupling biochemical and cellular methods in a screen/counter screen strategy might increase the specificity of these HTS assays.

## 7. Small molecule inhibitors of pre-mRNA splicing

**Figure 2.**
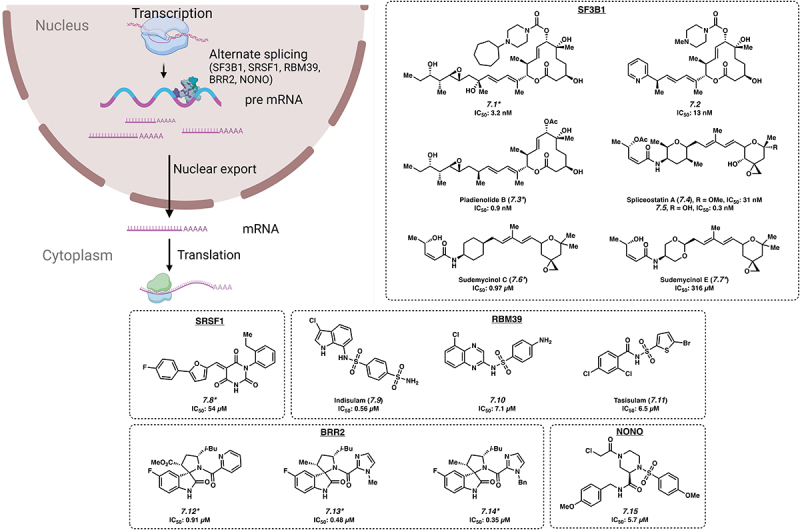
Small molecule inhibitors of pre-mRNA splicing. *Mechanism of action for inhibition not reported. Please see text for further details.

Splicing of pre-mRNA into mRNA is a fundamental process in eukaryotic cells and contributes to normal cellular functioning (Figure 2). However, aberrant splicing of pre-mRNA is a well-recognized feature of cancer, and one potential mechanism is the upregulation of malignant transcripts [48,49]. The proteins that control splicing, the splicing factor RBPs, are frequently mutated or otherwise dysregulated in cancer [13,50]. This has provided the impetus to develop small molecules to target these RBPs. Here, we highlight the role RBPs have in the increased translation of oncogenic transcripts and the development of small molecules to inhibit the effects of splicing factor RBPs.

### 7.1. SF3B1

SF3B1 is a core component of spliceosomes, the machinery that regulates splicing in eukaryotic cells. In many haematologic malignancies, SF3B1 mutations are thought to be ‘driver mutations’ and result in globally dysregulated splicing patterns. The mechanism is thought to involve alternative splicing of key oncogenic mRNAs, altering mRNA stability and protein isoform expression [51]. Hence, many therapeutic strategies have attempted to specifically inhibit this splicing factor. Small molecules E7107 (**7.1**) [52,53], H3B–8800 (**7.2**) [53], Pladienolide B (**7.3**) [53], Spliceostatin A (**7.4**) [54], FR901464 (**7.5**) [54] and Sudemycinol C (**7.6**) and E (**7.7**) [55] have been reported to bind to SF3B1. Notably, it was expected that Spliceostatin A (**7.4**) and **7.5** would bind in a covalent manner via the enone warhead; however, it was found that this binding occurred in a non-covalent manner (Figure 2) [54]. Growth inhibition of non-haematopoietic cell lines with **7.1**, **7.3**, **7.4** and **7.5** occurred at IC_50_ values of 3.2 nM, 0.9 nM, 31 nM and 0.3 nM, respectively [53,54]. These four compounds are thought to bind to SF3B1 and interfere with pre-mRNA splicing, both normal and mutant. Of these, **7.1** entered clinical trials, although it was discontinued due to side effects [56]. Another compound, **7.2** was shown to directly inhibit the SF3B1 complex by interfering with the interaction between the SF3b complex and the splicing branchpoint region. **7.2** was active against leukaemia cell lines at an IC_50_ of 13 nM and showed preferential killing of spliceosome-mutant cells, which the authors attribute to retention of short GC-rich introns, which are found in genes encoding splicing components [57]. This may provide an explanation for H3B–8800’s (**7.2**) preferential killing of spliceosome-mutant tumour cells, which are already deficient in splicing, and the killing effects were not attributable to the expression of a single target in the mutant cells. This compound is currently in ongoing clinical trials for myeloid malignancies [58]. Most recently, chemical proteomic screens have identified small molecules that can engage the SF3B1 protein, but the specificity for spliceosome mutant cells remains to be established [59]. Other components of the spliceosomal complex, such as SRSF1 have also been targeted by other groups (**7.8**), see ref [60].

### 7.2. RBM39

RBM39 was identified as a key member of a network of RBPs that were critical for maintaining RNA splicing and cell survival in acute myeloid leukaemia (AML) [61]. Indisulam (**7.9**) is an anti-tumour agent that acts as a molecular glue by recruiting the CUL4-DCAF15 ubiquitin ligase, resulting in polyubiquitination and proteasomal degradation of RBM39 [62]. Although it is active in inhibiting cell growth in both haematopoietic and non-haematopoietic cells [63], **7.9** demonstrated limited clinical efficacy in several trials, which were ultimately discontinued [64]. Analogs of **7.9**, CQS (**7.10**) and Tasisulam (**7.11**), use a similar mechanism to target RBM39 with IC_50_ values of 7.1 μM and 6.5 μM, respectively [62], but their utility remains to be determined.

### 7.3. BRR2

Mutations in SNRNP200/BRR2, a Ski2-like RNA helicase that appears to be a key component of the U5 SNRNP complex, are associated with retinitis pigmentosa [65], a form of degenerative blindness. Although its relevance in cancer and leukaemia remain unclear, several compounds have been developed to inhibit Brr2 including **7.12** with an IC_50_ value of 0.91 μM along with analogs **7.13** and **7.14** with IC_50_s of 0.48 μM and 0.35 μM, respectively [66].

### 7.4. NONO

The RBP NONO was discovered to be an androgen receptor-interacting protein which supports its transcriptional activity. The androgen receptor (AR) is a targetable oncogenic driver of prostate cancer; however, aberrant splicing of AR-encoding mRNA can lead to resistance against available treatments. NONO also interacts with a range of other cancer-specific genes, such as *SAMHD1*, and its expression may be pathogenetic in leukaemia and related to therapy resistance [67–69]. The search for inhibitors that deplete the AR led to the development of small molecule SKBG01 (**7.15**), with an IC_50_ value of 5.7 μM in HEK cells. This compound covalently modifies C145 of NONO, altering its binding and regulation of target transcripts, ultimately reducing the quantity of AR. C145 of NONO is located in a hinge region flanked by two RNA recognition motifs, and proximal to two residues important for RNA binding. Furthermore, there is evidence that **7.15** and related compounds act by stabilizing the interaction between NONO and its bound mRNAs. Ultimately, this stabilization effectively stalls transcript processing and maturation, resulting in decreased amount of target mRNA. This represents a unique mechanism of activity (i.e. functional modification rather than inhibition), shown by the authors to be dependent on the presence of wild-type NONO and the covalent modification of the protein by **7.15** [67].

## 8. Epitranscriptomics: small molecules targeting RNA modifying enzymes

**Figure 3.**
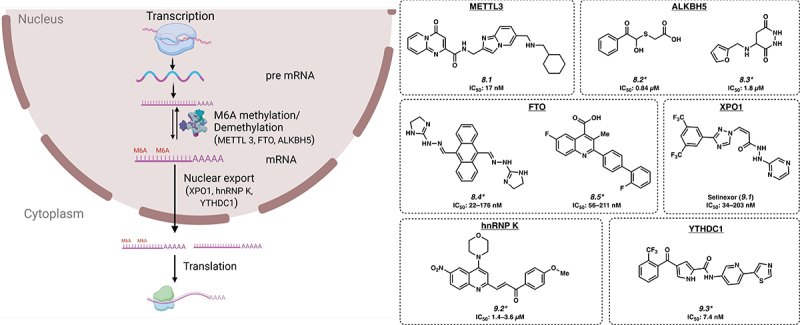
Inhibitors of RNA modification and nuclear export. *Mechanism of action for inhibition not reported. Please see text for further details.

The most prevalent modifications found on RNA molecules are m^6^A, m^1^A, m^5^C and m^7^G. These modifications play an important role in the gene regulation that affects various biological processes in cells (Figure 3). Among these, m^6^A (N^6^-methyladenosine) is the most abundant modification found on mRNA and has been the most widely studied modification in development and cancer. In acute myeloid leukaemia (AML), m^6^A modification is more prevalent on RNA and is required for the survival of the cancer cells [70], with a similar pattern noted in both leukaemia as well as in solid tumours [71]. Catalyzing the installation of a methyl group at the 6 position of adenosine to generate m^6^A are so-called epitranscriptomic writers; a second set of enzymes are known as erasers, which remove this modification.

### 8.1. METTL3

The RNA methylase enzymes METTL3 and METTL14 install the m^6^A modification and serve as writers in leukaemia cells. Recent work has led to the identification of STM2457 (**8.1**) as a small molecule inhibitor of METTL3 with an IC_50_ of 17 nM, with both in vitro and in vivo activity against AML cells via competitive binding with the SAM co-factor [72]. Many other small molecule inhibitors of METTL3 have been developed, which are mostly analogues of **8.1** and proceed through the same mechanism of action [73].

### 8.2. FTO/ALKBH5

On the other hand, m^6^A erasers include FTO and ALKBH5, two RBPs that catalyse the removal of m^6^A marks and play key roles in AML [17,74]. By removing the m^6^A marks, these two enzymes alter the epitranscriptome, and modify the stability of a number of crucial mRNA molecules such as *TACC3*, *ASB2* and *RARA* [17,74]. Compound III and IV (**8.2** and **8.3**) are small molecule inhibitors of ALKBH5 with IC_50_ values of 0.84 and 1.8 µM, respectively [75]. CS1 and CS2 (**8.4** and **8.5**) are small molecule inhibitors of FTO with IC_50_ values of 22–176 nM and 56–211 nM, respectively, across multiple cell lines [76]. However, the results of these studies are somewhat contradictory, as inhibition of both m^6^A writers and erasers have been shown to have activity in leukaemia [17,72–74]. Further work is required to clarify how the m^6^A mark, and its modulation, may represent an effective therapeutic strategy in leukaemia and in cancer.

## 9. Controlling nuclear export with small molecules

Nuclear export of mRNAs is a key step that allows for the timely and appropriate levels of gene expression to occur in the cytoplasm (Figure 3). Inhibitors of nuclear transport, specifically of XPO1 have shown clinically relevant activity in several cancers, notably in multiple myeloma [77]. Specifically, Selinexor (**9.1**) has been approved in combination therapy for multiple myeloma by the FDA and is being currently investigated in this context as well as for other haematologic malignancies. Selinexor (**9.1**) acts by covalently binding to XPO1 in the nuclear export signalling binding groove at C528, thus inhibiting nuclear export of mRNAs [78]. However, it is thought that additional RBPs may regulate nuclear export in a more nuanced manner. For example, heterogeneous nuclear ribonucleoproteins (hnRNPs) are a class of RBPs that binds to and regulate the nuclear export of the pre-mRNA. There are several different hnRNPs (A-U), and each protein appears to be selectively expressed in specific tissue types. hnRNAP K is overexpressed in human AML and experimental overexpression caused myeloproliferative disease in mice [79]. Compound 5 (**9.2**) is a small molecule inhibitor developed against hnRNP K with an IC_50_ of 1.4–3.6 µM [80]. Another RBP involved in mRNA transport, YTHDC1 appears to play a role in cancer [81] and is inhibited by YL-5092 (**9.3**) with an IC_50_ of 7.4 nM by binding to the m^6^A binding site (preprint) [82]. Other proteins such as ELAVL1/HuR and IGF2BP1 have also been reported to play roles in nuclear transport (see section **11.E**).

## 10. Small molecule inhibitors of the microRNA biogenesis pathway

**Figure 4.**
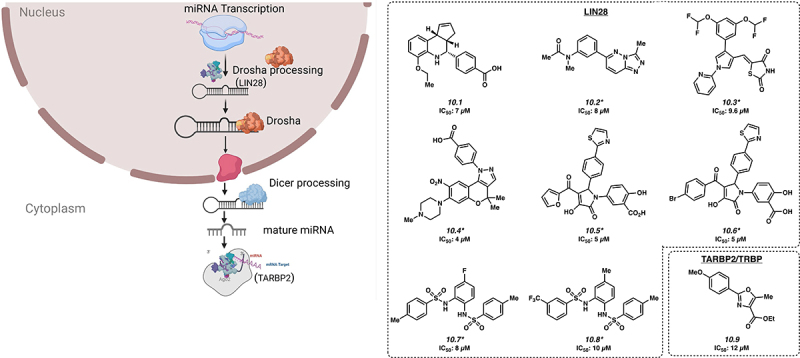
Inhibitors of microRNA processing. *Mechanism of action for inhibition not reported. Please see text for further details.

MicroRNA biogenesis is another pathway that is highly regulated by RBPs (Figure 4). Of note, one of the most studied miRNA regulators is LIN28, which is being targeted for the development small molecules against several cancer types.

### 10.1. LIN28

LIN28 consists of two paralogs, LIN28A and LIN28B, which are overexpressed in approximately 15% of human cancers, with overexpression generally associated with a poor prognosis [83]. LIN28 binds to precursor miRNA encoding the tumour suppressive let-7, preventing its maturation [84–86]. Small molecule LI71 (**10.1**) was discovered to have an IC_50_ of 7 μM in HeLa cells and targets LIN28 by binding to the CSD domain [87]. Similarly, Compound 1632 and KCB170552 (**10.2**, **10.3**) have shown inhibitory effects by interacting with LIN28 with IC_50_s of 8 μM and 9.6 μM, respectively [43,88,89]. Other small molecules that inhibit activity of the LIN28A protein include SB1301, PH-43, C902, CCG-233094 and CCG-223095 (**10.4** [41], **10.5** [43], **10.6** [43], **10.7** [47], and **10.8** [47] with low micromolar IC_50_s.

### 10.2. TARBP2

The RBP TARBP2 is a critical component of the RISC-loading complex, which allows for the loading of microRNAs and miRNAs into RISC, a key step in post-transcriptional gene regulation. This protein has been reported to be overexpressed or otherwise dysregulated in a number of cancer types [90]. CIB-3b (**10.9**) is a small molecule that binds to TARBP2, inhibiting the RISC-loading complex (IC_50_ of 12 µM) by disruption of the TRBP/Dicer interaction [91]. Inhibition of the RISC-loading complex ultimately reduces cancer cell proliferation by altering the expression of several different miRNAs processed by Dicer, which presumably result in the de-repression of cell proliferation inhibitory protein expression.

## 11. Small Molecule targeting of RNA stability modifiers

**Figure 5.**
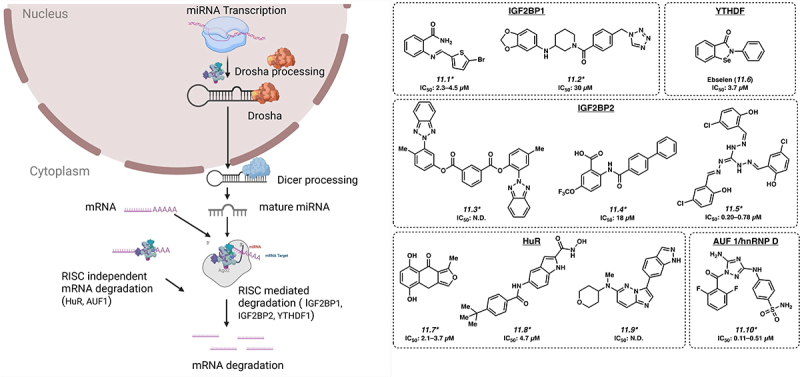
Small molecules targeting regulators of mRNA stability. *Mechanism of action for inhibition not reported. Please see text for further details.

RBPs can regulate stability of mRNAs, through the interpretation of epitranscriptomic marks, or via regulation of other mechanisms of RNA stability, such as RISC (Figure 5). These do not reflect mutually exclusive categories, with m^6^A reader proteins including the IGF2BP proteins and the YTHDF proteins [92]. RBPs reported to interact with and regulate RISC include the IGF2BP proteins, as well as HuR/ELAVL1, and hnRNP family of proteins. In the context of leukaemia, these targets include a number of oncogenic mRNA transcripts, such as *LIN28B*, *HMGA2* and *ZFP36L1* [25,93].

### 11.1. IGF2BP proteins

There are three proteins in this family: IGF2BP1, IGF2BP2 and IGF2BP3. In polar cells such as fibroblasts and neurons, IGF2BP1 appears to participate in the localization of mRNA transcripts such as *ACTB* [94]. However, it is also reported to bind to m^6^A-modified mRNA and participate in post-transcriptional gene regulation at the level of regulating mRNA stability and/or translation, thereby playing a role in oncogenesis [95]. BTYNB and 7773 (**11.1** and **11.2**) are two small molecule inhibitors developed against IGF2BP1 with IC_50_s of 2.3–4.5 and 30 µM, respectively, and show activity in a variety of solid tumour cell lines, via regulation of critical oncogenes such as MYC, RAS and E2F [96–98]. The IGF2BP2 protein, also a m^6^A reader, was recently shown to play an important role in acute myeloid leukaemia pathogenesis by regulating critical mRNA transcripts that in turn control glutamine metabolism [19]. Three compounds have been reported to have activity against IGF2BP2: small molecule inhibitors JX-5 (**11.3;** IC_50_: not disclosed) [99], Compound 4 (**11.4**; IC_50_: 18 µM) [100] and CWI1–2 (**11.5**; IC_50_: 0.20–0.78 µM) [19]. IGF2BP3 is overexpressed in 15% of human cancers, including KMT2A-translocated acute leukaemia [26,27]. An isocorydine derivative, d-ICD is reported to downregulate IGF2BP3 expression, although direct targeting of IGF2BP3 has not been reported [101].

### 11.2. YTHDF proteins

YTHDF proteins represent another class of RBPs that are cytosolic m^6^A readers that regulate the degradation and translation of m^6^A decorated mRNA. YTHDF2 regulates the degradation of m^6^A modified mRNA [102] and is inhibited by small molecule inhibitor Ebselen (**11.6**) with an IC_50_ value of 3.7 µM [103]. **11.6** forms a covalent Se – S bond with YTHDF at C412, nearby the m^6^A-binding pocket of YTHDF1. While this residue is not in the m^6^A pocket, binding of **11.6** inhibits the conformational rearrangement of the β4–β5 loop into the m^6^A binding-competent conformation necessary for interaction with m^6^A-labelled RNA.

### 11.3. ELAVL1/HuR

ELAVL1/HuR is reported to be dysregulated in leukaemia, lymphoma and a range of other cancer types, generally showing overexpression [23,104]. This is a multifunctional protein that plays a role in several mechanisms of RNA homoeostasis. In addition to a role in mRNA localization, under stress conditions, HuR interacts with miR-19 to relieve the suppression of *RhoB* mRNA to proceed for translation [105]. HuR has been reported to function in a RISC independent manner by binding to AU-rich elements (ARE)-containing mRNA, leading to the degradation of the mRNA. Small molecule inhibitors targeting HuR are MS-444 (**11.7**; IC_50_ of 2.1–3.7 µM), 1c (**11.8;** IC_50_ of 4.7 µM) and SRI-42127 (**11.9**; IC_50_: not disclosed) [106–108]. The mechanism of action is not entirely clear for these molecules.

### 11.4. AUF1/hnRNP D

AUF1/hnRNP D under stress condition binds to ARE-containing mRNA and may play an important role in degradation of oxidized mRNA [109]. JNJ-7706621 (**11.10**), a small molecule inhibitor targeting AUF1, has been described with an IC_50_ of 0.11–0.51 µM [110].

## 12. Targeting protein translation and/or mRNA sequestration

**Figure 6.**
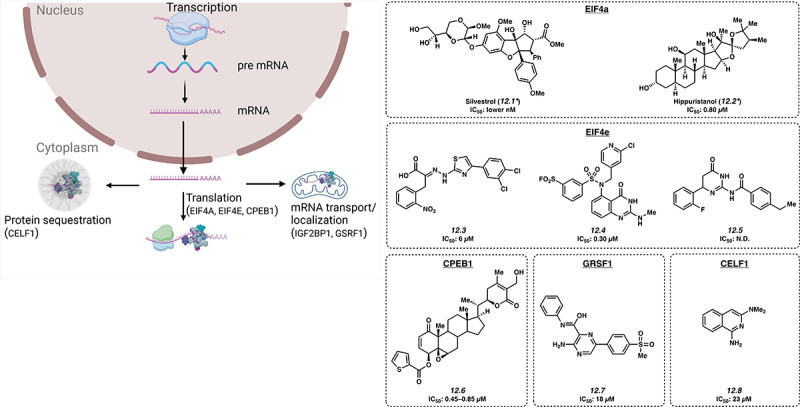
Inhibitors of protein translation and sequestration. *Mechanism of action for inhibition not reported. Please see text for further details.

The net effect of transcriptional and post-transcriptional mechanisms discussed thus far is to regulate the amount of mRNA that is accessible to the ribosome for translation into protein (Figure 6). mRNA may be freely available to the ribosome, or may be sequestered into various cellular compartments, preventing translation. Here, we review selected RBPs that regulate these steps of RNA homoeostasis.

### 12.1. EIF4A

eIF4A is a member of the DEAD-box family on RNA helicases. In combination with translation factors, it readies mRNA templates during translation initiation. High levels of eIF4A are seen in cancers such as T-cell acute lymphoblastic leukaemia (T-ALL), gastric cancer and cervical cancer with a proposed role via promotion of oncogenic mRNA translation [111,112]. The first small molecule inhibitor of eIF4A reported to have activity in T-cell ALL was natural product Silvestrol (**12.1**), with a low nanomolar IC_50_ value [111]. Another small molecule inhibitor of eIF4A, Hippuristanol (**12.2**) has been found to inhibit eIF4A with an IC_50_ of about 0.80 µM in HeLa cells [113].

### 12.2. EIF4E

Increased levels of eIF4E correlate with the growth of many types of tumours. The eIF4E/eIF4G complex is important in regulating gene expression via transcription initiation. The complex is regulated by 4E-BPs, known for their tumour-suppressor activity. By mimicking 4E-BP activity, small molecule 4EGI–1 (**12.3**) binds to eIF4E and interrupts eIF4E/eIF4G interaction with an IC_50_ of 6 μM in A549 cells by inhibiting cap-dependent translation [114]. Another small molecule inhibitor of EIF4E is Compound 12 (**12.4**) with an IC_50_ of 0.30 µM [115], which binds covalently between the sulphonyl group and K162, also inhibiting cap-dependent translation. Notably, **12.4** was identified using HTS in silico docking. Bn7GxP-based PROTACs (see section 13 for discussion of chemical proximity induced agents) also inhibited eIF4E at high micromolar concentrations [116]; however, these compounds showed minimal effect in cellular assays likely due to limited cell permeability. Small molecule 094 (**12.5**; IC_50_: not disclosed) was also found to inhibit the EIF4E-RM38 complex via inhibition of the protein-protein interaction [117].

### 12.3. CPEB1

CPEB1 is a translation regulator linked to increased metastatic potential in various cancer types including bladder cancer by downregulating TWIST1 and inducing EMT. A variant in the gene is thought to influence risk of chronic lymphocytic leukaemia [118]. Small molecule ASR488 (**12.6**) has been shown to bind with CPEB1 leading to cell growth arrest and cell death with an IC_50_ of 0.45–0.85 µM in TCCSUP cells [119].

### 12.4. GRSF1

GRSF1 is important in most steps of post-transcriptional gene regulation. It has an important part in cancer progression, and is thought to cause metastasis of cervical cancer through the PI3K/AKT/NF-κB and TIMP3/MMP9 pathways. Levels are also observed to be increased in patients with hepatocellular carcinoma (HCC). Studies using VE-821 (**12.7**), a small molecule, developed to inhibit GRSF1, yielded results showing an IC_50_ of 18 μM in Hep3B cells. It appears to act by decreasing GRSF1-dependent YY1 stability, a transcriptional activator of oncogenes, potentially by competing with miR-30e-5p microRNA-mediated repression [120].

### 12.5. CELF1

The RNA binding protein, CUGBP Elav-like family member 1 (CELF1) binds to mRNAs to regulate splicing, translation, and decay and may thereby have a role in translational activation of epithelial-mesenchymal transition genes that drive tumour progression. A small molecule, Compound 27 (**12.8**), competitively binds CELF1, preventing GU-rich RNA complexation with an IC_50_ of 23 μM [121].

## 13. Proximity-based approaches to small molecule development

In recent years, the use of chemical induced proximity via chimeric small molecules has been hailed as a potentially ground-breaking approach in drug development. The first of these, protein- or proteolysis-targeting chimeras (PROTACs), represent hetero-bifunctional molecules that degrade proteins by targeting a specific protein for ubiquitin-proteasome mediated degradation, which is a highly conserved and central pathway for protein degradation. This was first described to target the methionine aminopeptidase MetAP-2, with the chimera consisting of a small molecule linked to a peptide in this early work [122]. Since then, numerous refinements of this technology have been reported, allowing for the tethering of two chemical moieties together, one targeting a protein of interest, and the other targeting an E3 ubiquitin ligase enzyme [123]. The recruitment of the E3 ubiquitin ligase results in the deposition of ubiquitin side chains on the protein, which is then targeted to the proteasome where it is degraded. In the RBP field, a recent report showed that this approach could be used to degrade SF3B1 [124], and as previously mentioned to target EIF4E [116]. For the latter strategy, the investigators designed 7-benzylguanosine analogs of the m^7^G cap, normally found on the 5’ end of mRNA molecules, linked to a E3 ubiquitin ligase recruiting small molecule. Another variation on this strategy is so-called RNA-PROTAC, where an RNA sequence is linked to a compound that binds to the ubiquitin ligase enzyme [125]. For sequence-specific RBPs (which may include several of the m^6^A readers), this strategy might be very useful. Lastly, recent work has led to the design of so-called RIBOTACs, where one portion of the chimeric molecule targets RNA molecules and the other portion recruits an RNA-degrading enzyme, such as RNAse L or RNAse H [126,127]. This latter approach was recently reported to be successful in degrading a microRNA, miR-155. The further development of RBP-binding small molecules could enable the recruitment of RNA-degrading enzymes as a targeted approach to degrade specific RNA ligands of RBPs. These chimeric approaches are likely to expand as the number of biological molecules with defined functions, other than ubiquitin ligases and RNAses, are targeted with small molecules. For example, a recent study reported a chimeric molecule (termed a transcriptional/epigenetic chemical inducer of proximity) capable of rewiring cancer-specific transcription by replacing epigenetic repression with transcriptional activation at specific genetic loci [128]. Such approaches hold promise to rationally design chimeric approaches against cancer-specific post-transcriptional gene regulation, as the biological mechanisms are better understood and small molecules binding specific RBPs are developed.

## 14. Conclusions

In the last 20 years, a remarkable amount of progress has been made in understanding the biology and pathology of RBPs. In haematologic malignancies, a number of important discoveries have led to targeting efforts, particularly with mutant splicing factors, and at least one small molecule remains in clinical trials. The recognition of m^6^A modifiers and readers and their importance in gene regulation is another major recent development in the field, and there are several new small molecules reported to target m^6^A writers and readers. A number of other promising small molecules are at various stages of development, and developments in screening and downstream development strategies promise to enrich the pipeline of small molecules. For example, small molecules can be transformed from binders to degraders of RBPs by the construction of heterobifunctional molecules. This field promises to expand rapidly, as it promises to bring novel biologically informed approaches to target proteins that were once deemed undruggable.

## Supplementary Material

Supplementary_Table_Structures.pdf
