# Supplementary material for: Small molecule inhibition of RNA binding proteins in haematologic cancer
Source: RNA Biol. 2024 Feb 8;21(1):1–14. doi: 10.1080/15476286.2024.2303558 (PMC10857685; doi:10.1080/15476286.2024.2303558)
Supplement: Supplementary_Table_Structures.pdf [file KRNB_A_2303558_SM2456.pdf]

Target	Name	Structure	IC_{50}	Ref.
SF3B1	E7107 (*7.1*)	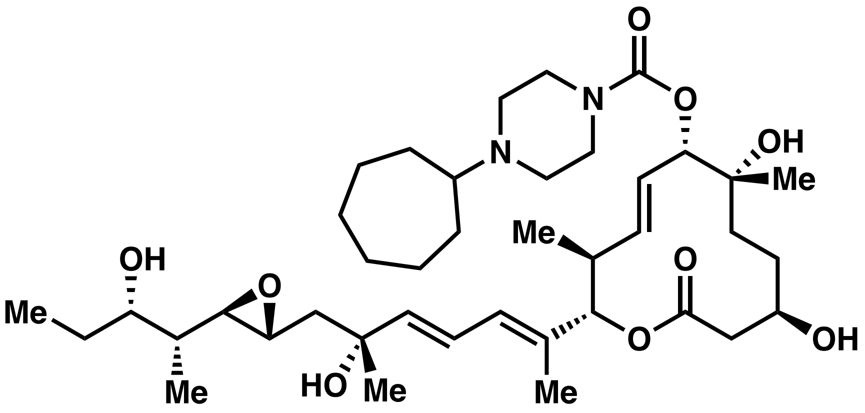	3.2 nM	52, 53, 56
	H3B-8800 (*7.2*)	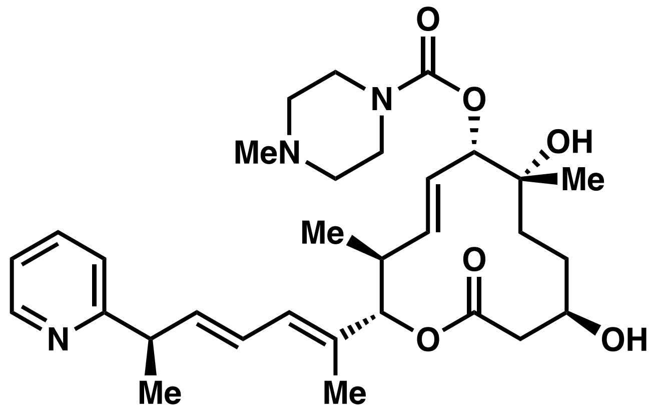	13 nM	57, 58
	Pladienolide B (*7.3*)	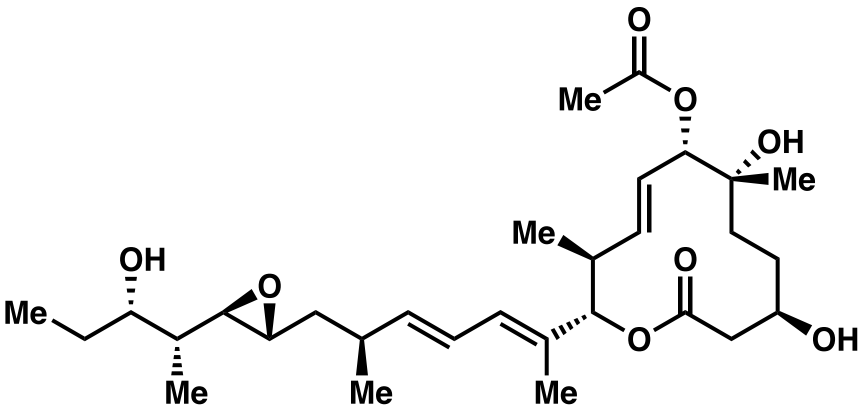	0.9 nM	53
	Spliceostatin A (*7.4*)	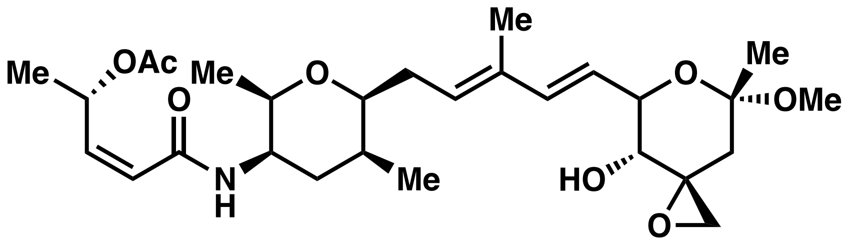	31 nM	54
	FR901464 (*7.5*)	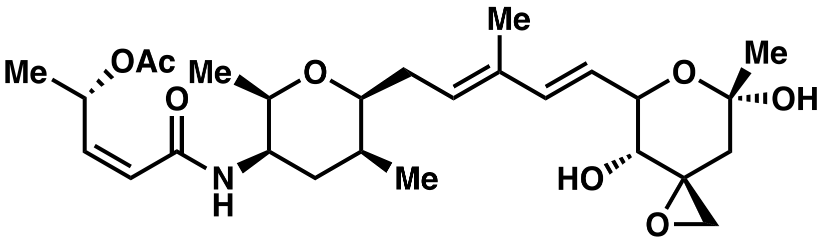	0.3 nM	54
	Sudemycinol C (*7.6*)	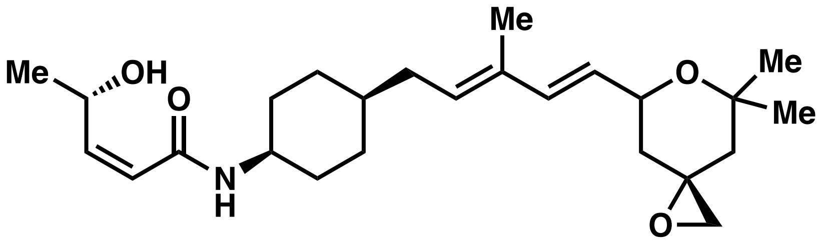	0.97 μM	55
	Sudemycinol E (*7.7*)	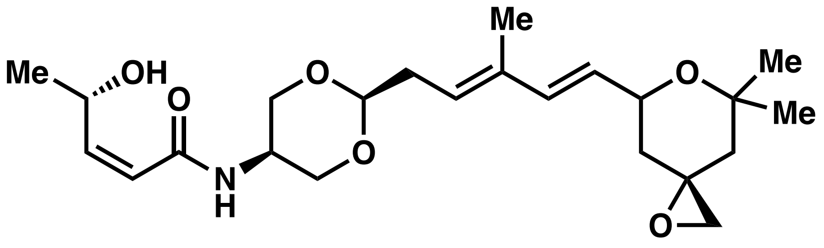	316 μM	55
SRSF1	cp028 (*7.8*)	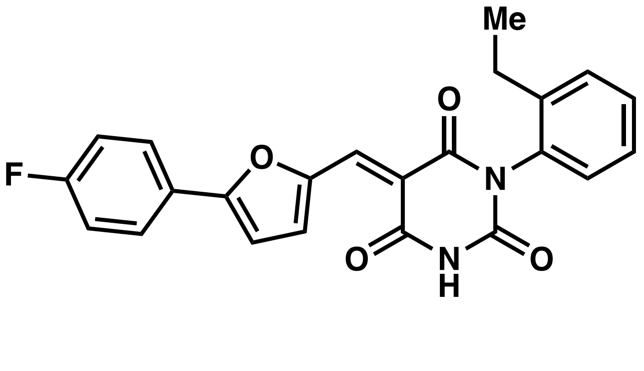	54 μM	60

Target	Name	Structure	IC_{50}	Ref.
	Indisulam (*7.9*)	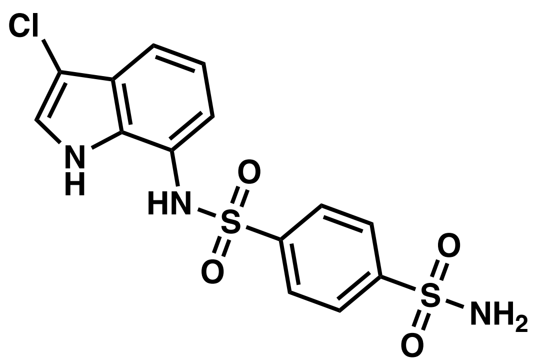	0.56 μM	62, 63, 64
RBM39	CQS (7.10)	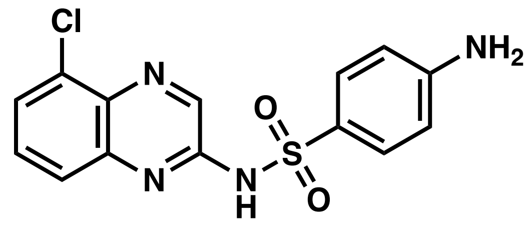	7.1 μM	62
	Tasisulam (*7.11*)	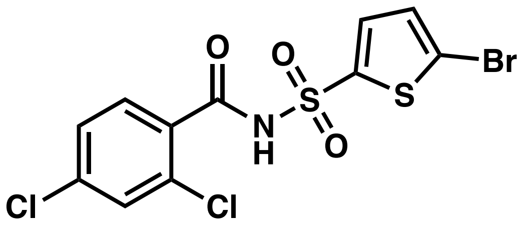	6.5 μM	62
	Compound 8 (*7.12*)	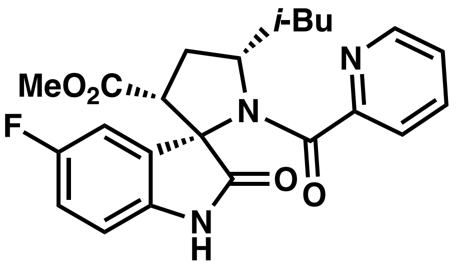	0.91 μM	66
BRR2	32a (*7.13*)	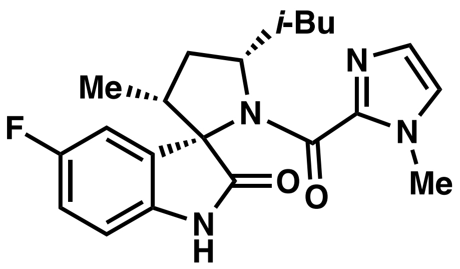	0.48 μM	66
	33a (*7.14*)	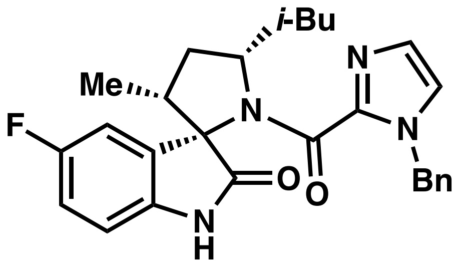	0.35 μM	66
NONO	SKBG01 (*7.15*)	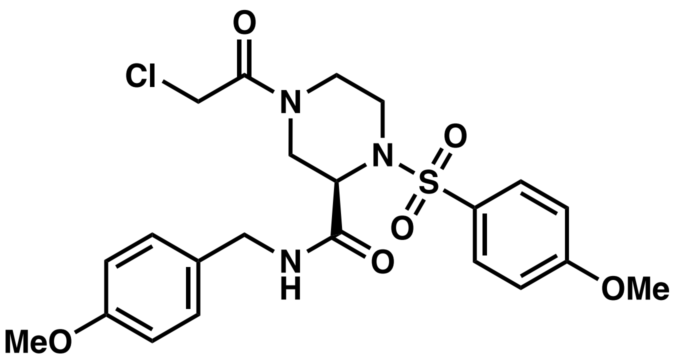	5.7 μM	67
METTL3	STM2457 (*8.1*)	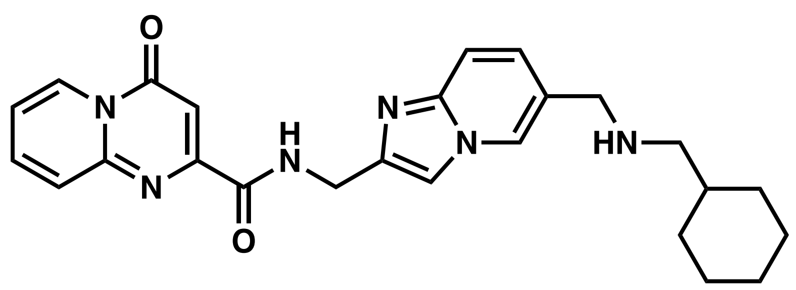	17 nM	72

Target	Name	Structure	IC_{50}	Ref.
ALKBH5	Compound III (*8.2*)	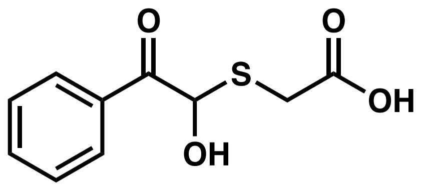	0.84 μM	76
	Compound IV (*8.3*)	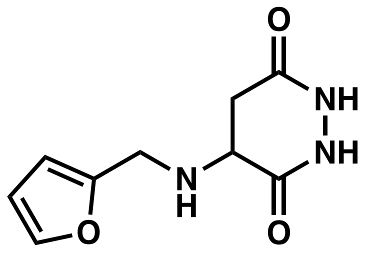	1.8 μM	76
FTO	CS1 (*8.4*)	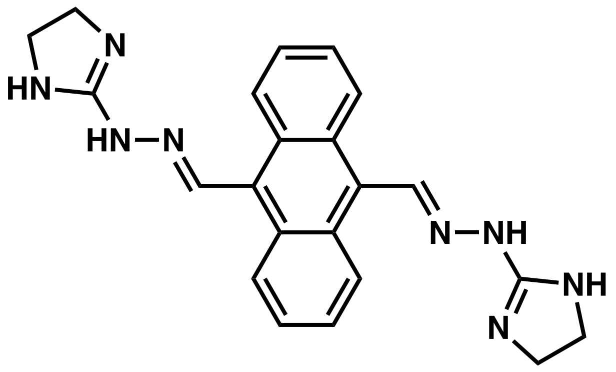	22–176 nM	77
	CS2 (*8.5*)	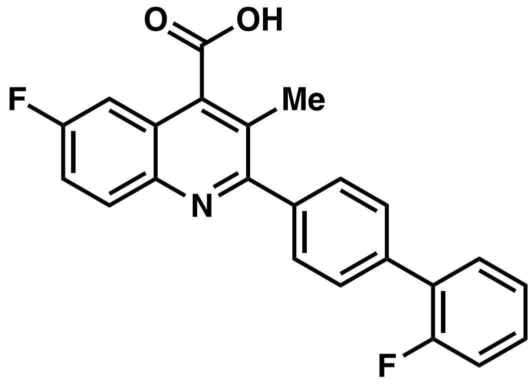	56–211 nM	77
XPO1	Selinexor (*9.1*)	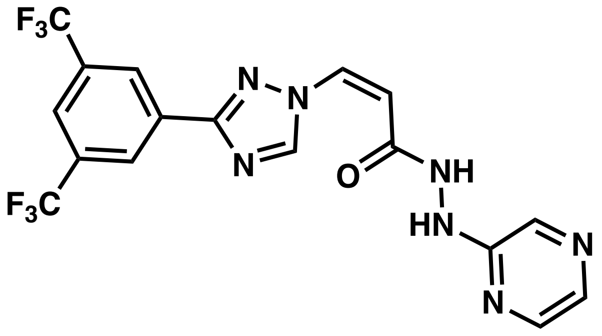	34–203 nM	79
hnRNP K	Compound 25 (*9.2*)	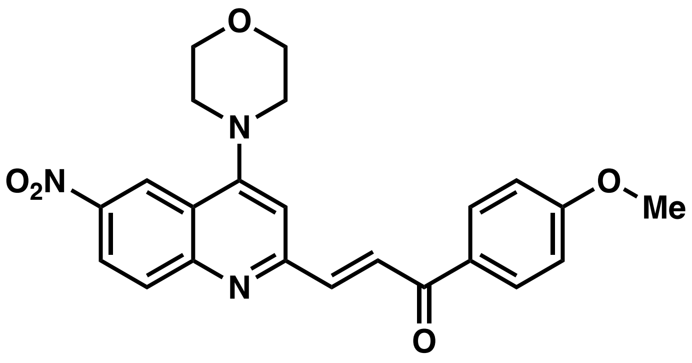	1.4–3.6 μM	81
YTHDC1	YL-5092 (*9.3*)	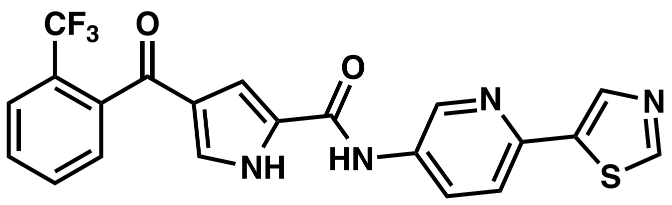	7.4 nM	83

Target	Name	Structure	IC_{50}	Ref.
LIN28	LI71 (*10.1*)	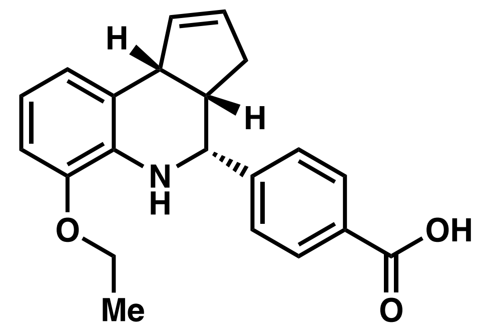	7 μM	88
	Compound 1632 (*10.2*)	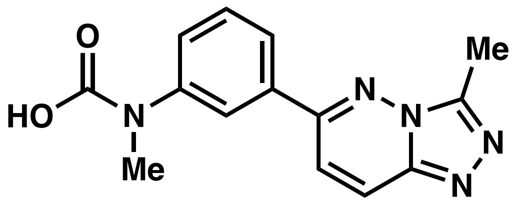	8 μM	43, 89, 90
	KCB170522 (*10.3*)	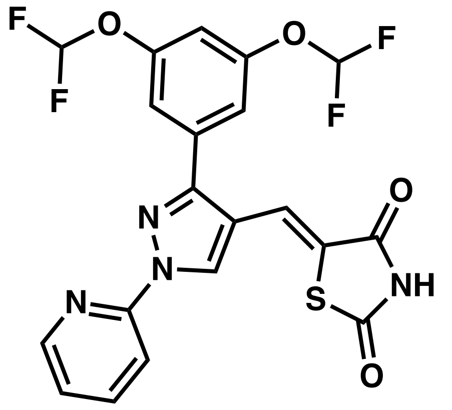	9.6 μM	43, 89, 90
	SB1301 (*10.4*)	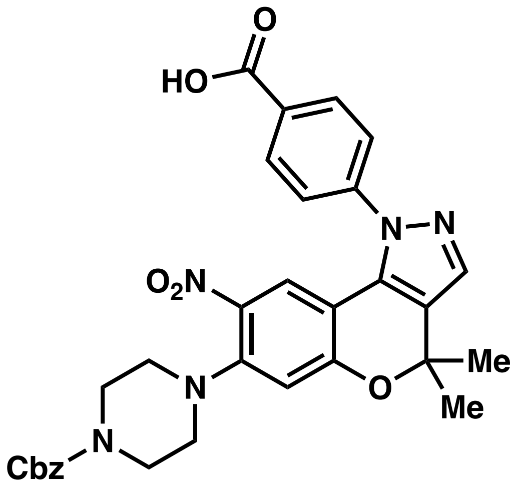	4 μM	41
	PH-43 (*10.5*)	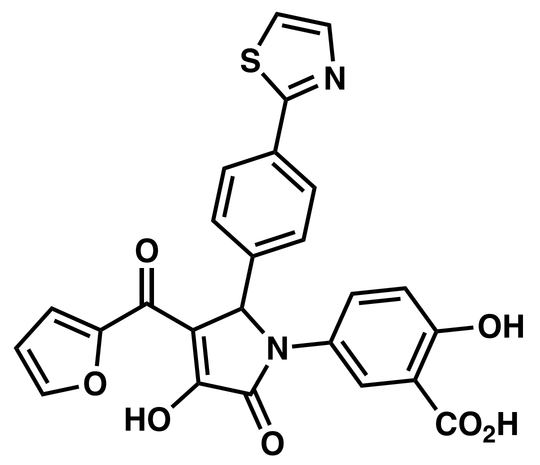	5 μM	43
	C902 (*10.6*)	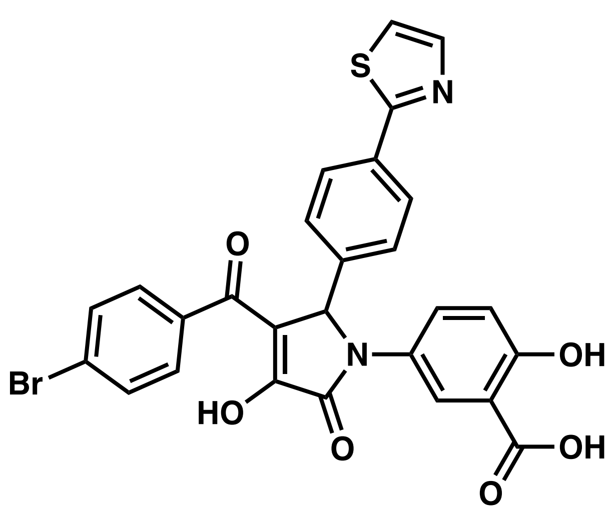	5 μM	43

Target	Name	Structure	IC_{50}	Ref.
LIN28 cont.	CCG-233094 (*10.7*)	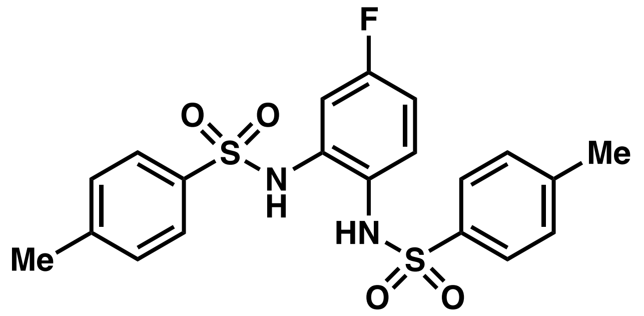	8 μM	47
	CCG-234459 (*10.8*)	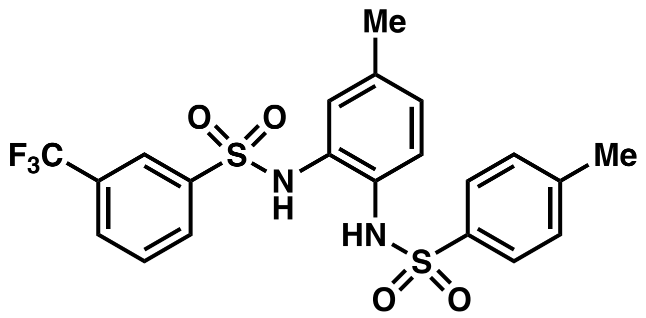	10 μM	47
TARBP2/TRBP	CIB-3b (*10.9*)	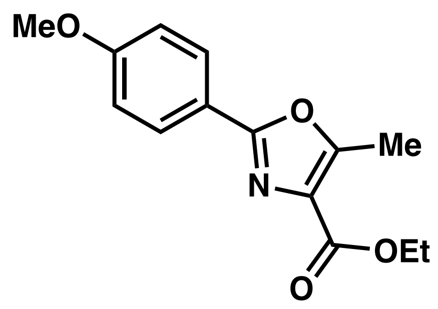	12 μM	92
IGF2BP1	BTYNB (*11.1*)	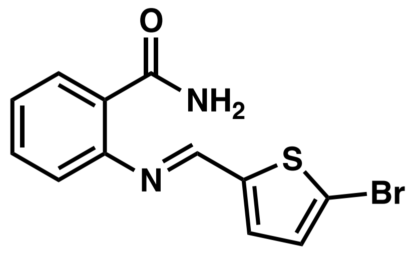	2.3–4.5 μM	97–99
	7773 (*11.2*)	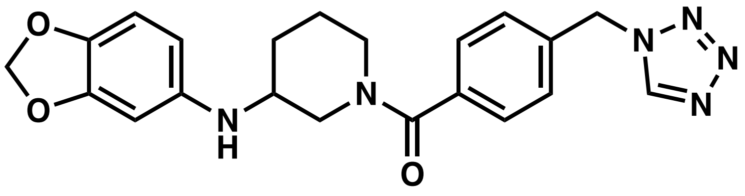	30 μM	98
IGF2BP2	JX5 (*11.3*)	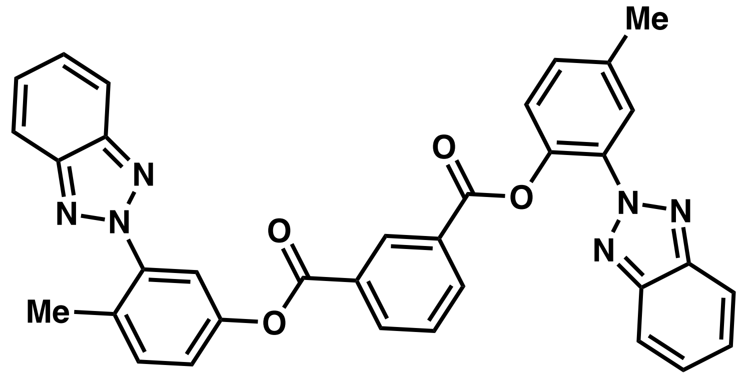	N.D.	100
	Compound 4 (*11.4*)	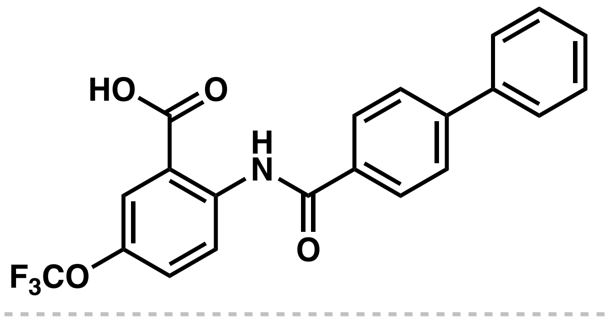	18 μM	101
	CWI1-2 (*11.5*)	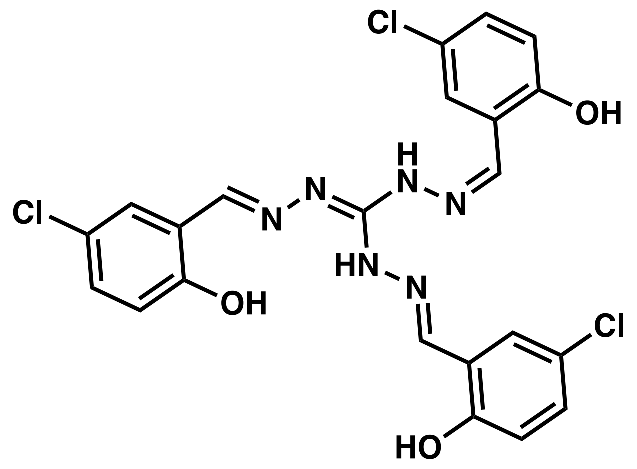	0.20–0.78 μM	19

Target	Name	Structure	IC_{50}	Ref.
YTHDF	Ebselen (*11.6*)	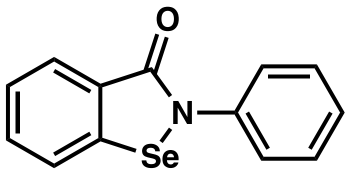	3.7 *μ*M	104
HuR	MS-444 (*11.7*)	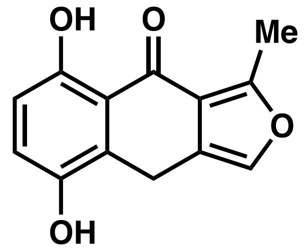	2.1–3.7 *μ*M	107
	1c (*11.8*)	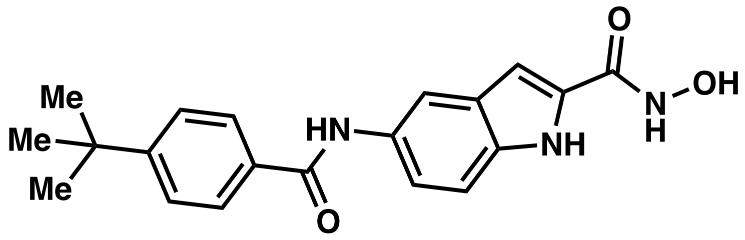	4.7 *μ*M	108
	SRI-42127 (*11.9*)	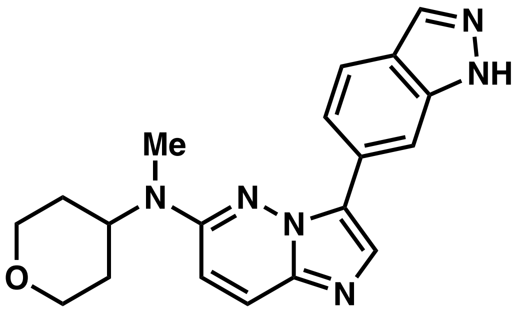	N.D.	109
AUF 1/hnRNP D	JNJ-7706621 (*11.10*)	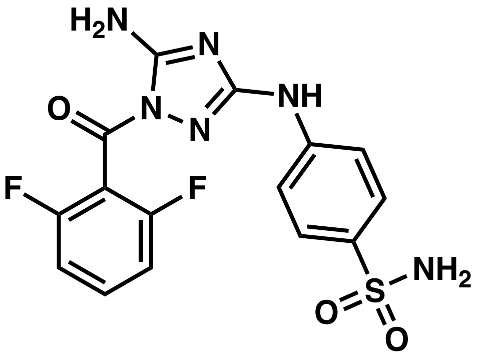	0.11–0.51 *μ*M	111
EIF4a	Silvestrol (*12.1*)	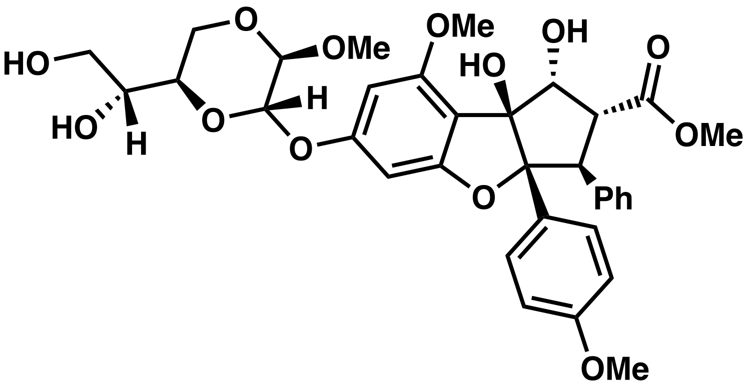	lower nM	112
	Hippuristanol (*12.2*)	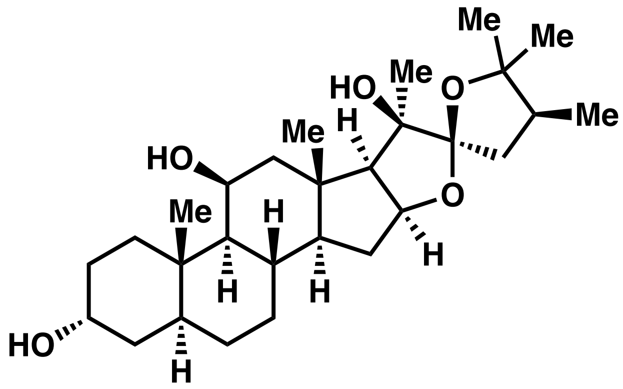	0.80 *μ*M	114
EIF4e	4EGI-1 (*12.3*)	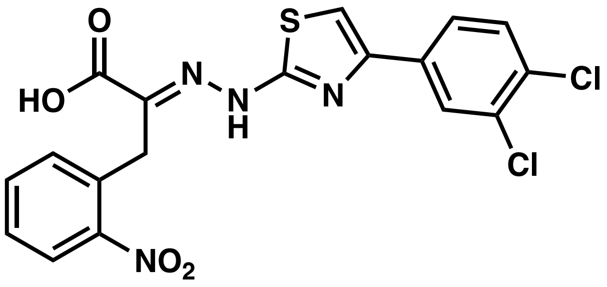	6 *μ*M	115

Target	Name	Structure	IC_{50}	Ref.
EIF4e cont.	Compound 12 (*12.4*)	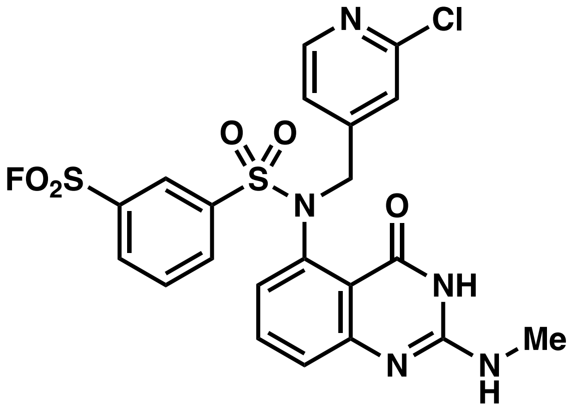	0.30 μM	116
	094 (*12.5*)	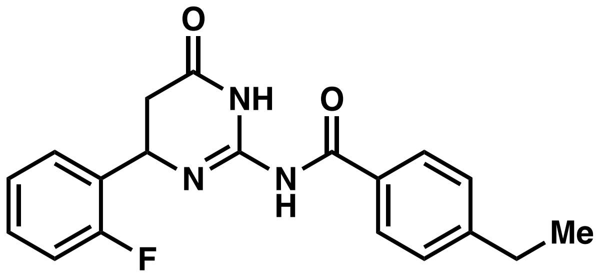	N.D.	118
CPEB1	ASR488 (*12.6*)	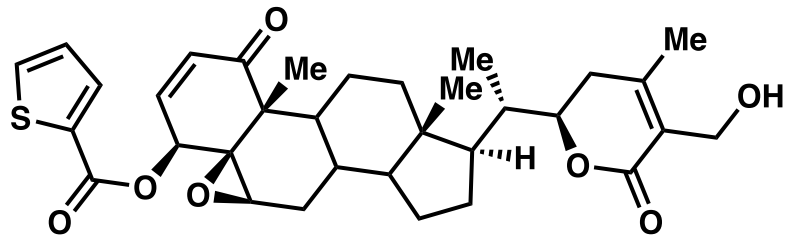	0.45–0.85 μM	120
GRSF1	VE-821 (*12.7*)	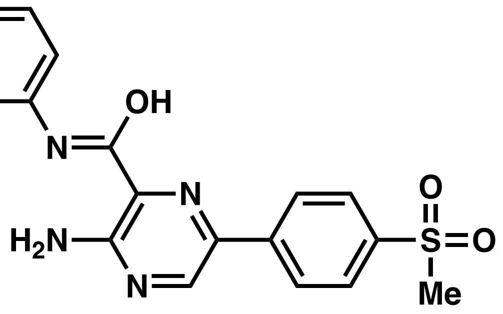	18 μM	121
CELF1	Compound 27 (*12.8*)	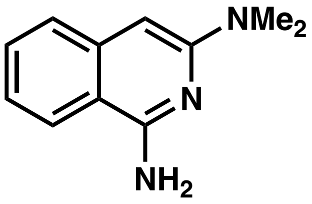	23 μM	122
